# Injection of Autologous Adipose Stromal Vascular Fraction in Combination with Autologous Conditioned Plasma for the Treatment of Advanced Knee Osteoarthritis Significantly Improves Clinical Symptoms

**DOI:** 10.3390/jcm13113031

**Published:** 2024-05-22

**Authors:** Markus Klingenberg, Antoniya Dineva, Annika Hoyer, Barbara Kaltschmidt, Philipp Leimkühler, Thomas Vordemvenne, Andreas Elsner, Dirk Wähnert

**Affiliations:** 1 Beta-Clinic Bonn, Joseph-Schumpeter-Allee 15, 53227 Bonn, Germany; 2 Biostatistics and Medical Biometry, Medical School OWL, Bielefeld University, Universitätsstrasse 25, 33615 Bielefeld, Germany; antoniya.dineva@uni-bielefeld.de (A.D.); annika.hoyer@uni-bielefeld.de (A.H.); 3 Department of Cell Biology, Bielefeld University, Universitätsstrasse 25, 33615 Bielefeld, Germany; barbara.kaltschmidt@uni-bielefeld.de; 4 Molecular Neurobiology, Bielefeld University, Universitätsstrasse 25, 33615 Bielefeld, Germany; 5 Department of Trauma and Orthopaedic Surgery, Medical School and University Medical Center OWL, Protestant Hospital of the Bethel Foundation, Bielefeld University, Burgsteig 13, 33617 Bielefeld, Germany; philipp.leimkuehler@evkb.de (P.L.); thomas.vordemvenne@evkb.de (T.V.); dirk.waehnert@evkb.de (D.W.); 6 DIOSS (German Institute for Orthopaedics, Osteopathy and Sports Medicine), Lipper Hellweg 10, 33604 Bielefeld, Germany; andreas.elsner@gmx.de; 7 Orthopedic Joint Practice at Bültmannshof, Kurt-Schumacher-Straße 17, 33615 Bielefeld, Germany

**Keywords:** knee osteoarthritis, stromal vascular fraction, orthobiologics, autologous adipose tissue, autologous conditioned plasma

## Abstract

(1) **Background:** Osteoarthritis (OA) is the most common joint disease in the world. It is chronic, systemic, progressive and disabling. Orthobiologics have the potential to positively alter the course of this disease. Therefore, the aim of this study is to evaluate the efficacy of SVF/ACP in the treatment of advanced osteoarthritis of the knee in an unfiltered patient population. We hypothesize that this therapy can improve the symptoms associated with osteoarthritis of the knee. We also hypothesize that there are patient-related factors that influence the efficacy of therapy. (2) **Methods:** Two hundred and thirteen patients with moderate to severe OA of the knee and SVF/ACP injection were recruited for this study. Patients were excluded if they did not provide informed consent or were not receiving SVF/ACP therapy. Pain, function, symptoms and quality of life were assessed using standardized scores (KOOS, WOMAC) before and after treatment. (3) **Results:** The VAS pain score was significantly reduced by at least 30% (*p* < 0.001). Knee function, as measured by the KOOS daily activity and sport scores, showed significant increases of 21% and 45%, respectively, at 6 months (*p* < 0.04). (4) **Conclusions:** Treatment of knee OA with SVF/ACP injection positively modifies the disease by significantly reducing pain and improving function.

## 1. Introduction

Osteoarthritis (OA) is the most common joint disease in Germany and worldwide [1]. Additionally, OA is an increasing, common and disabling disease that represents a substantial and growing health burden with significant implications for individuals, healthcare systems and broader socioeconomic costs [2,3].

Clinically, the knee is the most common site of osteoarthritis, accounting for approximately 85% of the global burden of osteoarthritis [4]. The advanced symptomatic osteoarthritis of the knee is a major health burden for patients due to pain and functional limitations that also affect social participation. In many cases, OA-related mobility limitations decrease patients’ ability to engage in daily physical activity and sports [2,5]. As a consequence, general exercise recommendations for the prevention or treatment of the most common lifestyle diseases, such as hypertension, metabolic disorders and obesity associated with sarcopenia, cannot be adequately followed [2].

**Figure 1:**
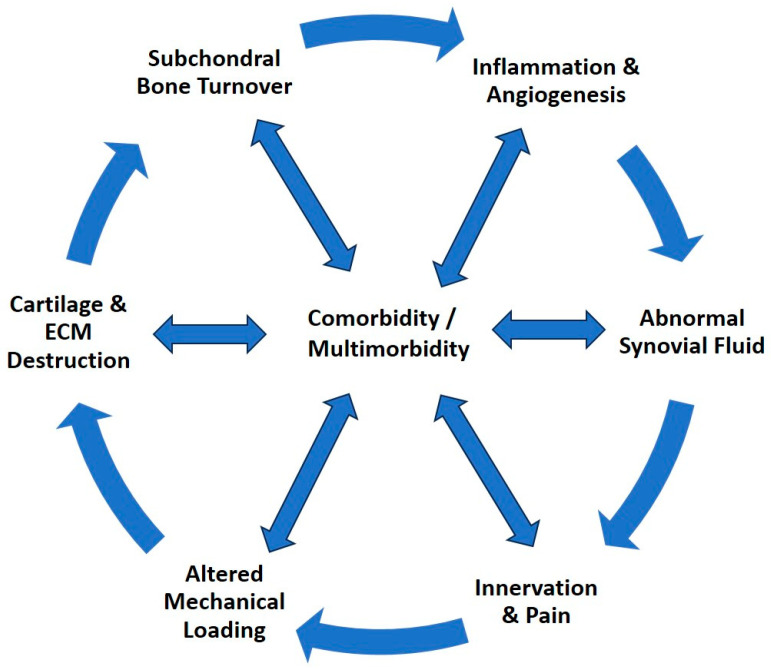
The progression cycle of osteoarthritis, a complex, systemic, chronic, progressive disease that affects and is affected by comorbidity and multimorbidity.

Osteoarthritis is a complex chronic progressive disease, often complicated by the presence of multimorbidity. In addition, physical inactivity, often due to pain, negatively affects existing comorbidities or increases the risk of such conditions, creating a vicious circle (Figure 1) [5,6].

Today, typical management is best characterized as palliative and reactive rather than focused on shared decision-making or coordinated and proactive and preventive interventions [5]. The guideline-based standard of care for OA of the knee in Germany includes local and oral analgesics, physical therapy, bracing, cortisone injections for acute inflammation and, in cases that cannot be adequately managed conservatively, arthroplasty [7]. In addition, the European Society for Sports Traumatology, Knee Surgery and Arthroscopy (ESSKA) recommends treatment with platelet-rich plasma (PRP) [8].

However, given the increasing individual and societal burden of osteoarthritis, the approach to management should change to individualized patient care based on specific needs [5]. Therefore, habit-building is crucial to minimize the frequency of recurrent activation of knee osteoarthritis. At best, this means a lifestyle intervention for patients.

A relatively new orthobiologic therapy approach is to use the stromal vascular fraction (SVF) of autologous adipose tissue and inject it together with autologous conditioned plasma (ACP) into the infra- or suprapatellar fat pad of the affected knee joint. SVF is derived from adipose tissue through mechanical processing. It contains several active cells and substances, such as pericytes, adipose-derived stem cells, and endothelial and progenitor cells. Mesenchymal stem cells, also found in SVF, are of particular interest and are also referred to as medicinal signaling cells. MSCs are known for their paracrine [9,10,11], anti-inflammatory [12,13] and immunomodulatory effects [14,15], as well as the secretion of various growth factors and cytokines [16,17]. Autologous conditioned plasma (ACP) is obtained from peripheral blood by centrifugation. The result is a leukocyte-poor platelet-rich plasma (PRP) [18].

The results of studies from recent years suggest that this therapy can also be used successfully for more severe osteoarthritis of the knee [19,20,21,22,23]. However, the number of cases in each study is small and/or the observation period is short [4,24,25,26,27]. In addition, the treatment methods and follow-up vary considerably [19,20].

Therefore, the aim of this study is to evaluate the efficacy of SVF/ACP in the treatment of advanced osteoarthritis of the knee in an unfiltered patient population. Pain and function will be monitored over a two-year follow-up period. In addition, success variables will be identified. We hypothesize that this therapy can improve symptoms associated with osteoarthritis of the knee. We also hypothesize that there are patient-related factors that influence the efficacy of this therapy.

## 2. Materials and Methods

### 2.1. Patient Cohort

For this monocentric retrospective study, all patients presenting to the study center from 2017 to 2023 with knee osteoarthritis and subsequent treatment with autologous adipose tissue-derived stromal vascular fraction (SVF) and autologous conditioned plasma were informed about the study and asked for consent. Inclusion criteria were as follows: 18 years or older, symptomatic OA of the knee, SVF/ACP therapy and informed consent. Patients with mechanical barriers, inadequate ROM for daily activities, joint instability or a pinching device such as a free joint body/meniscus, anticoagulation and active malignant processes were excluded from the study. A large number of participants had at least discussed joint replacement as a treatment option prior to this therapy. A total of 42% of participants reported regular use of pain medication in their medical history, 66% reported use of a brace, and 21% reported previous surgery on the affected knee. In addition, many had been treated with hyaluronic acid, cortisone and various plasma preparations as intra-articular injections. Thus, this study represents a non-selected, real-world cohort of patients. Data collection and management was performed using the Arthrex Surgical Outcomes System (SOS, Arthrex, Naples, FL, USA). Due to the closure of this database in the summer of 2023, not all participants have yet reached the 1- and 2-year follow-up. Therefore, of the 213 enrolled participants, 158 reached the 1-year follow-up, and 73 reached the 2-year follow-up. Of these, we had a dropout rate of 13% (138/158) at 1 year and 22% (57/73) at 2 years. The reason for dropout (e.g., unwillingness to continue participation or change in therapy to arthroplasty) was not recorded. This resulted in the study population shown in Table 1. The mean age is 62.5, varying slightly between females (62.1) and males (61.9). Most patients have severe (grade 4) and moderate (grade 3) osteoarthritis. For the majority of the study participants, one (41.8%) or two (50.2%) joints were treated in the overall course of the study. An additional booster therapy was applied to 33.3% of the study participants after 12 months.

Within the SOS platform, standardized information and patient-related outcome measures were collected at defined time points. The system automatically generated email invitations for follow-up appointments. Follow-up was performed at 2 and 6 weeks, 3 and 6 months, and 1 and 2 years after surgery. The VAS pain scale (0—no pain to 10—maximal pain) and the Knee Injury and Osteoarthritis Outcome Score (KOOS) with its 5 dimensions (pain, symptoms, activity of daily living, function and quality of life) were used. Each dimension of the KOOS score is calculated and scored independently, resulting in a total of 5 different scores for each dimension. The possible scores range from 0 to 100 points. A score of 100 indicates no limitations due to the affected knee, while a score of 0 indicates extreme problems/limitations. In addition, the KOOS JR score was used, which ranges from 0 to 100, with 0 representing total knee disability and 100 representing perfect knee health. In addition, the Western Ontario and McMaster Universities Osteoarthritis Index (WOMAC) represents the dimensions of pain, stiffness and physical function. We used a norming process of the scores so that higher scores indicate better outcomes and allow comparison with the KOOS. The SANE (Single Assessment Numeric Evaluation) for the knee was also used for the presented patient cohort. This is a single-question PROM for assessing joint limitation. Patients indicate the functionality of the joint by providing a score between 0% (non-functional) and 100% (normal functionality).

### 2.2. Intervention

All participants in this study presented to the study center with symptomatic osteoarthritis of the knee(s). When conservative treatment was exhausted, they were offered injection therapy using autologous adipose tissue stromal vascular fraction (SVF) and autologous conditioned plasma (ACP).

The Autologous Conditioned Adipose Tissue (ACA) technique (Arthrex, Naples, FL, USA) was used to prepare the SVF/ACP injection material. The ACA technique is a simple, cost-effective and rapid solution for harvesting and processing adipose tissue to obtain adipose-derived stromal adipose tissue. ACA-SVF is isolated by mechanical separation of adipose tissue. The preparation followed the manufacturer’s detailed instructions, which can be found at https://www.arthrex.com/de/weiterfuehrende-informationen/LI2-00040-EN/production-of-microfat?referringteam=orthobiologics, accessed on 21 March 2024. Depending on the joints affected by symptomatic osteoarthritis, one or two knee joints received the injection therapy. Because osteoarthritis is a systemic disease, some patients also received injections in other symptomatic joints. This was recorded as a covariate. In this study, the amount of abdominal fat harvested per joint treated was standardized to 30 mL. Due to inter-individual differences in fat composition, the amount of SVF obtained from these 30 mL varies from 2 to 6 mL. The ACP was prepared from 15 mL peripheral blood by centrifugation according to the manufacturer’s recommendations. This resulted in a variable amount of ACP ranging from 4 to 6 mL. After preparation, the SVF/ACP was injected into the infra- or suprapatellar fat pad. In a number of patients, a second injection was given after 12 months as a booster treatment, following the same procedure.

### 2.3. Data Acquisition and Statistical Analysis

Data were collected from the SOS database via an export to Excel (Microsoft Excel 365, Redmond, WA, USA). Raw data were processed by extracting sociodemographic variables (sex and age), relevant scores (VAS pain, KOOS, WOMAC and SANE) and additional risk factors that were included in the statistical analysis. Therefore, we considered the application of booster therapy and additional pain medication, the number of overall treated joints, and the osteoarthritis severity grade given by the Kallgren–Lawrence classification. To compare the pre- and post-treatment scores and to further evaluate their course over multiple measurement occasions, we used score-specific linear mixed models (LMMs). The aforementioned risk factors were included as explanatory covariates. Additionally, latent class linear mixed models (LCLMMs) [28] were used to identify groups of therapy responders and non-responders and to examine associations of the risk factors on the respective pain scores in the sub-groups. For all models, we performed a complete case analysis. Therefore, the measurements in the second year after intervention were not included due to a large number of missing values. *p*-values ≤ 0.05 were considered statistically significant.

The data analysis was performed using the statistical software R (version 4.3) [29].

## 3. Results

Table 2 shows the results of the descriptive analysis for the outcome measures, i.e., the VAS, WOMAC and KOOS score per measurement occasion. The results indicate an increase in the WOMAC and KOOS scores at the first follow-up, which either remains constant or further increases over the entire follow-up period. The VAS pain score declines after receiving the treatment and remains constant until the end of the follow-up.

**Figure 2:**
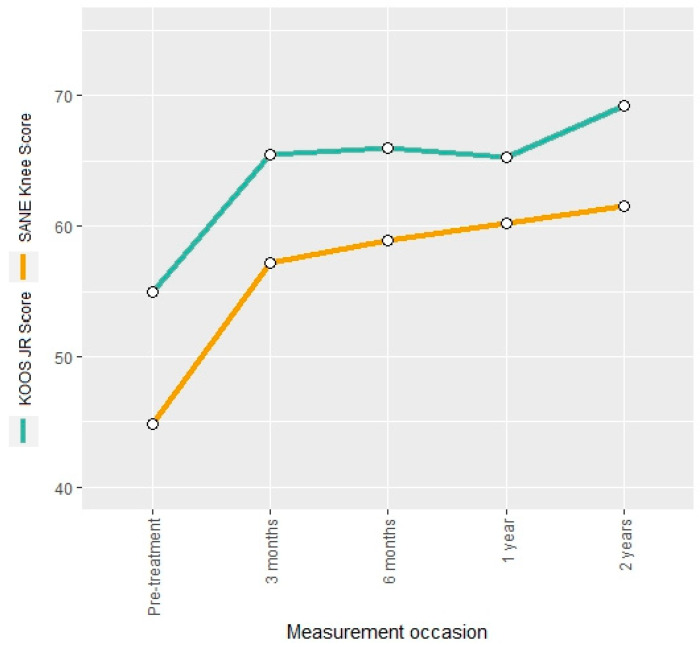
Mean KOOS JR score and SANE knee score at different measurement occasions.

The KOOS JR score and the SANE knee score were evaluated as general knee scores. Both measures showed an increase of 19% and 27%, respectively, at the three-month follow-up (Figure 2). The overall increase after two years was 26% and 37% for the KOOS JR score and the SANE knee score, respectively, without noticeable declines in between, indicating a constant improvement in the patient’s pain perception.

### 3.1. Pain

**Figure 3:**
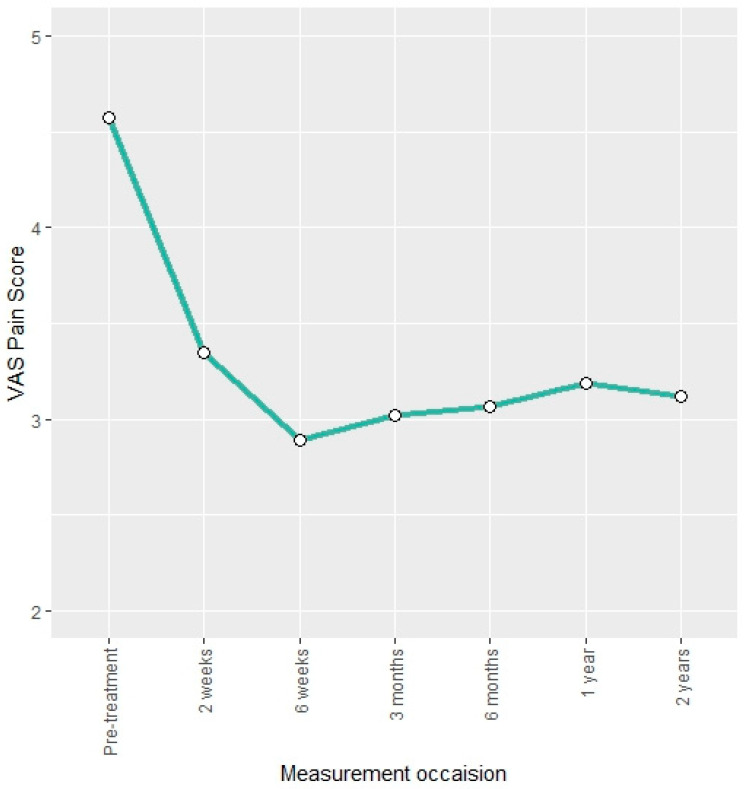
Mean VAS pain score at different measurement occasions.

The VAS pain score was reduced by approximately 34% at the three-month follow-up compared to pre-treatment. This reduction remained stable over the two-year follow-up (Figure 3). Descriptive findings were confirmed by the LMM as indicated by negative regression coefficients that are statistically significant (Table 3).

Similar results were found for the KOOS pain score and WOMAC pain score, which showed an increase of 26% and 23%, respectively, after the intervention. This trend remained constant throughout the two-year follow-up. The estimates of the respective score-specific LMMs indicated a significant increase at all but the last post-interventional measurement occasion (Tables S1 and S2 in Supplementary Material).

Parameters associated with the pain scores in the entire cohort included age, sex, number of joints treated, booster therapy and grade of OA (Table 3). In general, positive regression coefficients indicate an increase in the score, whereas negative values point to a decline in the outcome. For the VAS pain score, we found that a one-year increase in age resulted in a 0.03 ([0.00; 0.05], *p* = 0.06) higher score. Male participants had, on average, a 0.17 ([−0.48; 0.81], *p* = 0.63) higher score compared to females. The application of a booster therapy decreased the mean score by −0.23 ([−0.87; 0.41], *p* = 0.5), and lower grades of OA showed lower mean scores (−0.65 [−3.94; 2.65], *p* = 0.71 for grade 2 and −0.17 [−1.14; 0.79], *p* = 0.74 for grade 3) compared to grade 4.

Furthermore, we assessed the influence of the different covariates in both sub-groups identified by the LCLMMs. The non-responder group (group 1) showed a noticeable increase in the pain score post-treatment (Figure S1 in Supplementary Material), whereas, for the responder group (group 2), a decline in the score after receiving the intervention was observed (Figure S1 in Supplementary Material). Model estimates for the responder group suggest that male patients had, on average, −0.2 [−0.72; 0.33] (*p* = 0.49) lower scores than females (Table S3 in Supplementary Material). Lower grades (grades 2 and 3) showed greater pain improvement compared to grade 4. In addition, the number of joints treated (as an indicator of OA activity) is a negative indicator, meaning that patients with multiple joints treated showed less improvement than patients with fewer joints treated (*p* = 0.09), as did BMI. Increasing BMI is associated with higher VAS pain score and hence less pain improvement (*p* = 0.05). Looking at the non-responder group, we found that male sex was a negative predictor, meaning that male patients showed less pain improvement than female patients did. The other variables showed inconsistent results when comparing pain scores (Table S4 in Supplementary Material).

Taken together, these findings suggest that this therapy may be more effective in reducing pain in young female patients with low BMI and low-grade osteoarthritis.

### 3.2. Symptoms

**Figure 4:**
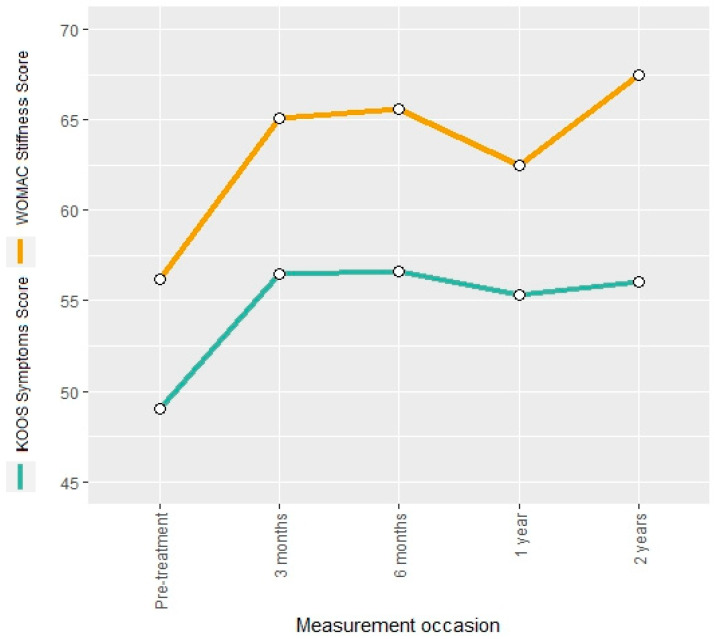
Mean KOOS symptoms score and WOMAC stiffness score at different measurement occasions.

The KOOS symptoms score and the WOMAC stiffness score were used to describe the evolution of osteoarthritis symptoms. Both measures showed an increase of 15% and 16%, respectively, after the intervention. The estimated regression coefficients for the mean change at the follow-up occasions indicated a significant increase for both scores (Tables S5 and S6 in Supplementary Material). The KOOS symptoms score remained constant over the entire follow-up period, whereas the WOMAC stiffness score showed a further increase of 20% at the 24-month follow-up compared to the pre-interventional measurement (Figure 4).

Covariates associated with the WOMAC stiffness score were age, BMI, number of treated joints and degree of OA. The results suggest a significant decline in the WOMAC stiffness score of −0.32 ([−0.59; −0.04], *p* = 0.03) points on average when increasing the age by one year. The increase in BMI results in a significant reduction in the stiffness score (−1.03 [−1.77; −0.28], *p* = 0.01), and the increase in one treated joint leads to a significant score decline (−5.46 [−10.62; −0.29], *p* = 0.05) (Table S6). In contrast, patients with grade 2 OA showed an average 36.49 [0.81; 72.17] points higher score compared to patients with grade 4 OA (*p* = 0.06). These findings were confirmed by the KOOS symptoms score for which the increase in the BMI is associated with a significant decline of −0.47 points ([−0.88; −0.06], *p* = 0.03), and the increase in one additionally treated joint results with a significant decrease (−4.61 [−7.44; −1.79], *p* < 0.001) (Table S4).

### 3.3. Function

**Figure 5:**
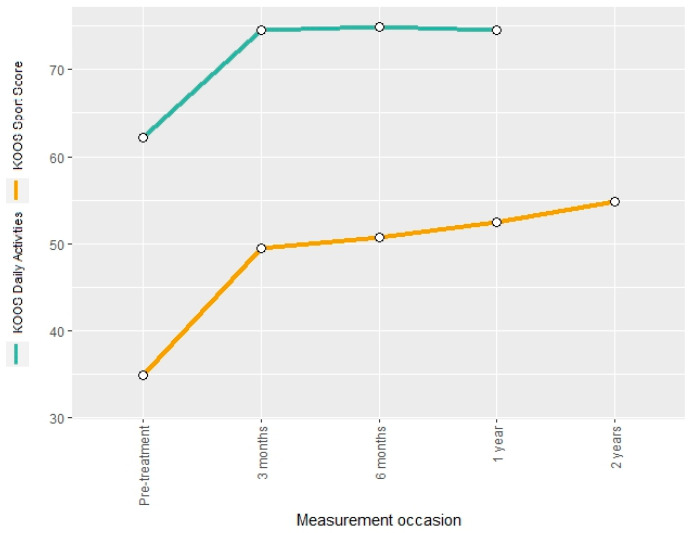
Mean KOOS sport score and KOOS daily activities score at different measurement occasions.

The KOOS daily activities score and the KOOS sport score were used to describe treatment-related changes in knee function. The two measures showed a post-interventional increase of 20% and 41%, respectively. The KOOS daily activities score remained constant until the end of the two-year follow-up, whereas the KOOS sport score showed a further increase of 57% in comparison to the pre-interventional measurement (Figure 5).

The respective estimates of the LMMs for the mean score changes at the follow-up time points indicate a significant increase at all but the last post-interventional measurement occasion (Tables S7 and S8).

Parameters with a statistically significant association with the KOOS daily activity score were age, BMI and the number of treated joints. The results indicated that an increase in age by one year is associated with −0.37 ([−0.59; −0.15], *p* < 0.001) points on average, and an increase in BMI by one unit reduces the score by −0.65 ([−1.25; −0.06], *p* = 0.04) points. The treatment of one additional joint is associated with an average decline of −4.69 ([−8.84; −0.54], *p* = 0.04). In contrast, applying a booster therapy results in a mean increase of 0.41 [−5.05; 5.88] points (*p* = 0.89). Patients classified with grade 2 OA demonstrate 10.36 ([−18.29; 39.02], *p* = 0.5) higher mean score than those with grade 4 OA. No statistically significant risk factors associated with the KOOS sport score were found, but the increase in one additional treated joint results in a mean score change by −5.77 ([−16.11; 4.57], *p* = 0.32) points, an application of booster therapy results in 11.69 [−0.69; 24.08] points (*p* = 0.1) and OA grade 2 results in 15.5 [−29.22; 60.22] higher mean score compared to grade 4 (*p* = 0.54).

### 3.4. Quality of Life

The KOOS quality of life score showed an increase of 52% after the intervention at the 3-month follow-up and kept accelerating, leading to an 81% increase at the last measurement occasion compared to the pre-intervention score. No statistically significant associations between the KOOS quality of life score and potential risk factors.

## 4. Discussion

This study presents 2-year results of orthobiologic therapy using SVF/ACP injection for moderate/severe osteoarthritis of the knee in a real-world patient cohort. This therapy can significantly reduce pain levels and improve functional outcomes throughout the follow-up period. However, patients can be divided into responders and non-responders. This study evaluated relevant patient-related factors that influence the success of therapy. Age, gender, BMI and booster therapy were identified as relevant patient factors. Overall, we demonstrated a 37% increase in the SANE knee score and an 81% increase in the KOOS quality of life score at two-year follow-ups.

Most importantly, we were able to demonstrate a clinically significant reduction in pain levels. The VAS pain score decreased by approximately 34% after treatment and remained constant over the 2-year follow-up. This was also true for the WOMAC pain score, which showed a 23% improvement after the intervention. According to the literature, these results can be classified as clinically relevant improvements in pain. In several studies, the minimum clinically meaningful difference for the WOMAC pain score in OA patients was determined, and a change of at least 10% to 22% was reported as clinically relevant [30,31].

As a disease-modifying therapy, SVF/ACP injection also had a positive effect on the development of OA symptoms, as shown by the KOOS symptoms and the WOMAC stiffness score. Both improved by approximately 15% after the intervention and remained at least constant over the 2-year follow-up. This change can also be considered relevant, as a substantial clinical benefit can be expected if the KOOS symptom score increases by at least 10.7 points [32] and the WOMAC stiffness score by 2.6 points [33].

The KOOS daily activity score and the KOOS sport score showed improvements of 20% and 41%, respectively. These scores remained constant or improved over the 2-year follow-up period. This change can also be considered relevant because the minimal clinically important difference for the KOOS daily activity + sport score is 25 points, and a substantial clinical benefit can be expected if the score increases by at least 30 points [32].

It is known that OA is mostly triggered by micro and macro injuries to the affected joints. The repair processes initiate proinflammatory immune cascades that eventually lead to progressive joint destruction. SVF is derived from adipose tissue, an attractive source of mesenchymal stem cells due to the abundance of adipose tissue and its easy accessibility. Another important factor is age, as OA is a disease of the elderly. Wu and coworkers studied the effects of age on human adipose-derived stem cells. They were able to show that the amount of adipose-derived mesenchymal stem cells per unit of lipoaspirate is maintained despite age, in contrast to decreasing amounts in bone marrow aspirate [34]. Specifically for application in the musculoskeletal system, Wu et al. concluded that adipose-derived MSCs exhibit similar osteogenic paracrine activity independent of age, and they suggest that the clinical applicability of adipose-derived mesenchymal stem cells is maintained regardless of age [34].

Significant chondrogenic effects from the application of adipose-derived mesenchymal stem cells have been demonstrated in in vitro studies. These studies suggest that SVF possesses the CD73, CD90, CD105 and CD106 markers, which are surface markers required for cell differentiation into cartilage [20,35,36,37]. In addition, and perhaps more clinically relevant, there is also a paracrine effect of SVF on OA chondrocytes by promoting inhibitory macrophages and T regulatory cells, which may reduce inflammatory markers and lead to pain relief and functional improvement, as also demonstrated in our study [20,36,37].

Studies have shown inconsistent results regarding the clinical relevance of cartilage regeneration after SVF treatment, ranging from significant cartilage regeneration [22,23,24] to no effect of SVF injection on cartilage [25,26,38,39]. One of the results of the MRI studies is more consistent—the reduction in bone marrow edema after the injection of SVF [27,38,40].

There is clinical evidence that SVF injection can significantly improve clinical outcomes such as pain and function in patients with OA of the knee, as shown in the systematic review by Aletto et al. [19]. Furthermore, the network meta-analysis by Anil et al. has shown that SVF injections are significantly more effective than several other injection therapies in improving clinical pain and function in patients with OA of the knee [20]. The main finding of this meta-analysis was that SVF resulted in the highest P-score for VAS pain at all time points. This indicates that SVF had the greatest effect on the development of pain after injection therapy at all time points. In addition, SVF had the highest WOMAC score 1 year after injection, indicating that these patients also had the highest functional outcome scores after treatment [20]. Mautner and colleagues compared cell-based injections with corticosteroid injections for knee pain in patients with osteoarthritis (Kellgren–Lawrence II–IV) [21]. They found that none of the three orthobiologic injections were superior to any other or to corticosteroids at 1 year after injection regarding VAS pain and KOOS pain scores [21].

Nevertheless, numerous treatment options exist for knee osteoarthritis, each with its own set of advantages and disadvantages. In order to facilitate the evaluation of the SVF/ACP therapy within this context, we conducted a comparative analysis of the outcome measures associated with different treatment options, as presented in Table 4.

SVF/ACP injection is an effective disease-modifying therapy that allows for treating multiple joints as well as repeated treatment of the same joint. Thus, this therapy has the potential to prolong the time of arthroplasty or even reduce the number of arthroplasties. In addition, and importantly, patients’ quality of life is improved by this therapy.

It is well known from clinical practice that every therapy has both responders and non-responders. In the present study, we evaluated factors that predict response. For the main clinical problem, pain, we found that SVF/ACP injection therapy may be more effective in reducing the level of pain in younger female patients with low BMI and lower grade (<4) osteoarthritis. Also, for the symptoms and function, we found younger age, lower BMI, fewer joints treated and lower degree of OA to be values that appear to have a positive effect on response to therapy. Additionally, booster therapy tends to have a beneficial influence on the function. The study by Yokota et al. compared the safety and efficacy of adipose-derived stromal cells (ASC) versus stromal vascular fraction (SVF) injection in patients with knee OA [46]. In the ASC group, patients whose VAS pain scores had decreased by less than 50% from baseline were given a booster injection after 6 to 12 months. They found no additional clinical benefit from the booster injection. However, the booster injections significantly increased the risk of injection site pain and swelling compared to the first injection [46]. Unfortunately, booster therapy was not performed in the SVF group.

SVF injection therapy is a safe and effective treatment for OA of the knee [19,20,47,48,49]. The procedure for preparing SVF is simple and requires minimal additional time. No serious adverse events have been reported in several studies [19]. Common adverse events include joint pain, hematoma and recurrent effusion [19].

SVF injection therapy has several other advantages, including the ability to treat multiple joints and the use of booster therapies. In addition, this therapy preserves the biological joint, and all other therapeutic options, such as arthroplasty, are still possible.

However, SVF injection therapy also has limitations. First of all, the comparability of the results of the clinical trials is limited due to differences in methodology and confounding factors [19,48]. In our cohort, we tried to reduce this by evaluating data from a single center and a single surgeon performing the procedures. The SVF preparation procedure was standardized and kept constant during the study period. Another limitation is the non-standard composition of the SVF. SVF is a mixture of pericytes, fibroblasts, adipocytes, monocytes, macrophages, red blood cells and ADSCs. However, the potency and concentration of the stem cell component cannot be evaluated or standardized in the setting used in our study. In their study, Garza et al. achieved superior results in symptoms and pain with a high dose of intra-articular SVF compared to a low-dose injection. Both high and low doses were superior to placebo [25]. Another important factor is the use of concomitant procedures such as arthroscopic debridement, microfracture, or high tibial osteotomy, which prevents a clear understanding of the true contribution and clinical potential of SVF treatment [19]. In our cohort, treatment was standardized, and none of these procedures were performed in the study cohort to overcome this limitation. Further relevant limitations of this study are the retrospective data analysis and the fact that not all patients were able to reach the 1- and 2-year follow-up due to the closure of the registry. Other limitations are that the evaluation of the SOS registry only reports PROMS, clinical evaluation was not possible, and the reason for dropout was not known.

Any orthobiologic therapy such as SVF/ACP injection for moderate to advanced osteoarthritis is a continuum because it is a chronic progressive disease. Therefore, this therapeutic option can be used in the recurrent phases of painful activation of osteoarthritis, in addition to nutrition (supplementation), exercise, bracing, etc. However, more research is needed to develop standardized treatment protocols. In the authors’ opinion, the following topics are of particular interest: first, individual boosters based on symptoms and/or time. On the one hand, too many injections in the context of continuous therapy should be avoided (risk of infection). On the other hand, further damage to the remaining cartilage should be reduced or even avoided. Boosters can generally be performed with ACP/SVF or hyaluronic acid. In the present study, the annual booster was used for standardization. In the meantime, our recommendation is as follows: booster at the earliest after three months if symptoms are still present; booster at the latest after 12 months regardless of symptoms. Of course, it would be even more accurate to measure the synovial fluid at different times to determine the degree of inflammation in the joint based on inflammatory parameters or mediators. Another factor is adipose tissue as a source of SVF. The authors are interested in the “quality” of the adipose tissue and if there are possibilities to precondition the patients/the adipose tissue. In addition, the role of modifiable comorbidities is still unclear. Furthermore, the optimal dose–response is unclear.

## 5. Conclusions

This study shows that SVF/ACP injection is an effective option for patients with OA of the knee and results in a significant reduction in pain levels. In addition, symptoms, function and quality of life improved considerably. We identified several parameters (e.g., age, BMI, degree of OA) as parameters influencing the response to therapy.

## Figures and Tables

**Table 1:** Descriptive analysis of the entire cohort. Continuous variables are given as mean (standard deviation) and categorical variables as frequency (proportion).

Variable	Overall (*n* = 213)	Female (*n* = 91)	Male (*n* = 122)
Age	62.0 (12.5)	62.1 (12.4)	61.9 (12.7)
BMI	27 (4.5)	26.8 (5.1)	27.1 (4.0)
Overall treated joints			
1	89 (41.8%)	32 (35.2%)	57 (46.7%)
2	107 (50.2%)	53 (58.2%)	54 (44.3%)
3	15 (7.0%)	4 (4.4%)	11 (9.0%)
4	2 (0.9%)	2 (2.2%)	0 (0.0%)
Booster			
No	142 (66.7%)	58 (63.7%)	84 (68.9%)
Yes	71 (33.3%)	33 (36.3%)	38 (31.1%)
OA (osteoarthritis) severity			
Grade 4	53 (24.9%)	22 (24.2%)	31 (25.4%)
Grade 3	157 (73.7%)	69 (75.8%)	88 (72.1%)
Grade 2	3 (1.4%)	0 (0.0%)	3 (2.5%)
Medications			
No	124 (58.2%)	50 (54.9%)	74 (60.7%)
Yes	89 (41.8%)	41 (45.1%)	48 (39.3%)

**Table 2:** Summary of results for patient-reported outcome measures as mean (standard deviation).

Variable	Pre-Treatment	3 Months	6 Months	1 Year	2 Years
VAS Pain Score	4.58 (2.32)	3.02 (2.36)	3.06 (2.45)	3.19 (2.55)	3.12 (2.56)
KOOS Pain Score	55.06 (16.82)	69.21 (16.84)	69.20 (19.24)	69.2 (19.59)	72.58 (18.87)
KOOS Symptoms Score	49.06 (13.76)	56.49 (12.67)	56.64 (13.07)	55.29 (12.68)	56.04 (14.64)
KOOS Daily Activities Score	62.16 (20.24)	74.56 (17.89)	74.91 (19.61)	74.45 (19.62)	78.98 (18.97)
KOOS Sport Score	34.96 (27.08)	49.44 (26.56)	50.72 (29.15)	52.48 (28.95)	54.79 (27.59)
KOOS Quality of Life Score	28.76 (17.12)	43.82 (20.25)	45.46 (22.04)	46.40 (22.97)	52.05 (23.82)
KOOS JR Score	54.97 (13.68)	65.44 (13.81)	65.99 (15.59)	65.33 (15.89)	69.21 (16.34)
WOMAC Pain Score	60.97 (19.04)	75.11 (17.94)	74.68 (19.74)	74.85 (20.15)	78.18 (20.53)
WOMAC Stiffness Score	56.19 (25.36)	64.05 (21.60)	65.99 (22.83)	62.50 (24.06)	67.5 (23.52)
SANE Knee Score	44.83 (20.35)	57.13 (22.61)	58.91 (23.82)	60.18 (24.63)	61.51 (24.62)

**Table 3:** Estimated model parameters for VAS pain score on the full cohort.

Variable	Regression Coefficients [95% Confidence Interval]	*p*-Value
Intercept	1.06 [−1.56; 3.69]	0.45
Measurement occasion 2 weeks ^1^	−1.09 [−1.53; −0.64]	<0.0001
Measurement occasion 6 weeks ^1^	−1.49 [−1.94; −1.05]	<0.0001
Measurement occasion 3 months ^1^	−1.28 [−1.72; −0.84]	<0.0001
Measurement occasion 6 months ^1^	−1.10 [−1.54; −0.65]	<0.0001
Measurement occasion 1 year ^1^	−1.01 [−1.45; −0.57]	<0.0001
Age	0.03 [0.00; 0.05]	0.06
Sex (male)	0.17 [−0.48; 0.81]	0.63
BMI	0.04 [−0.03; 0.11]	0.30
Duration of symptoms (months)	0.01 [−0.01; 0.02]	0.37
Number of treated joints (overall)	0.34 [−0.14; 0.81]	0.19
Booster (yes)	−0.23 [−0.87; 0.41]	0.50
Osteoarthritis severity (Grade 2) ^2^	−0.65 [−3.94; 2.65]	0.71
Osteoarthritis severity (Grade 3) ^2^	−0.17 [−1.14; 0.79]	0.74
Medications (yes)	−0.15 [−0.80; 0.51]	0.68

^1^ Reference group: pre-treatment measurement. ^2^ Reference group: osteoarthritis severity (Grade 4).

**Table 4:** Treatment options for knee osteoarthritis. Values are mean improvement values. (m: months; w: weeks; HA: hyaluronic acid; NSAIDs: nonsteroidal anti-inflammatory drugs; ESWT: Extracorporeal Shockwave Therapy).

	Follow-Up Time	VAS Pain Absolut	VAS Pain %	KOOS Absolut	KOOS %	WOMAC Absolut	WOMAC %
SVF/ACP (own data)	12 m	1.4	30%	10.1	18%	11.4	31%
HA [41]	11 m	3.4		18.7		25.3	45%
HA [42]	48 w	2.5	52%	13.8	20%	12.8	17%
NSAIDs [42]	48 w	1.8	38%	9.7	14%	11.0	15%
ESWT [42]	48 w	2.0	57%	14.0	19%	13.8	18%
Steroids [43]	6 m	0.5	10%			6.6	14%
HA [44]	3 m	0.8	11%			3.57	7%
Steroids [44]	3 m	0.4	5%			3.21	6%
Physiotherapy [45]	6 m	4.5	65%	34.2	73%	32.3	64%

## Data Availability

The data presented in this study are available on request from the corresponding author.

## Supplementary Materials

The following supporting information can be downloaded at: https://www.mdpi.com/article/10.3390/jcm13113031/s1, Table S1: Estimated model parameters for WOMAC pain score on the full cohort. Table S2: Estimated model parameters for KOOS pain score on the full cohort. Table S3: Estimated model parameters for VAS pain score in Group 2 (responder group). Table S4: Estimated model parameters for VAS Pain score in Group 1 (non-responder group). Table S5: Estimated model parameters for KOOS Symptoms score on the full cohort. Table S6: Estimated model parameters for WOMAC stiffness score on the full cohort. Table S7: Estimated model parameters for KOOS daily activities score on the full cohort. Table S8: Estimated model parameters for KOOS sport score on the full cohort. Figure S1: Individual trajectories of VAS pain score for therapy non-responders (Group 1) and therapy responders (Group 2).
