# Supplementary material for: Injection of Autologous Adipose Stromal Vascular Fraction in Combination with Autologous Conditioned Plasma for the Treatment of Advanced Knee Osteoarthritis Significantly Improves Clinical Symptoms
Source: J Clin Med. 2024 May 22;13(11):3031. doi: 10.3390/jcm13113031 (PMC11172752; doi:10.3390/jcm13113031)
Supplement: Supplementary file 1 [file jcm-13-03031-s001.zip › jcm-2952680-supplementary.pdf]

Supplementary Material

Table S1. Estimated model parameters for WOMAC Pain score on the full cohort.

Variable	Regression coefficients [95% confidence interval]	p-value
Intercept	104.28 [82.26; 126.31]	< 0.001
Measurement occasion 3 months[1]	12.59 [9.14; 16.04]	< 0.001
Measurement occasion 6 months[1]	12.97[9.52; 16.42]	< 0.001
Measurement occasion 1 year[1]	11.98 [8.53; 15.43]	0.23
Age	-0.25 [-0.47; -0.04]	0.03
Sex (Male)	1.31 [-4.02; 6.65]	0.64
BMI	-0.71 [-1.29; -0.14]	0.02
Duration Symptoms (Months)	-0.05 [-0.16; 0.06]	0.41
Number of treated joints (overall)	-4.97 [-8.98; -0.96]	0.02
Booster (yes)	0.10 [-5.19; 5.39]	0.97
Osteoarthritis severity (Grade 2)[2]	8.00 [-19.71; 35.71]	0.59
Osteoarthritis severity (Grade 3)[2]	0.59 [-7.49; 8.66]	0.89
Medications (yes)	2.90 [-2.57; 8.37]	0.32

[1] Reference group: Pre-treatment measurement. [2] Reference group: Osteoarthritis severity (Grade 4).

Table S2. Estimated model parameters for KOOS Pain score on the full cohort.

Variable	Regression coefficients [95% confidence interval]	p-value
Intercept	91.00 [69.86; 112.14]	< 0.001
Measurement occasion 3 months[1]	12.84 [9.81; 15.87]	< 0.001
Measurement occasion 6 months[1]	13.86 [10.83; 16.90]	< 0.001
Measurement occasion 1 year[1]	11.95 [8.92; 14.98]	0.23
Age	-0.25 [-0.46; -0.04]	0.02
Sex (Male)	1.60 [-3.52; 6.72]	0.56
BMI	-0.58 [-1.13; -0.03]	0.05
Duration Symptoms (Months)	-0.03 [-0.14; 0.08]	0.61
Number of treated joints (overall)	-3.91[-7.77; -0.06]	0.06
Booster (yes)	-0.09 [-5.17; 4.98]	0.97
Osteoarthritis severity (Grade 2)[2]	8.00 [-19.71; 35.71]	0.59
Osteoarthritis severity (Grade 3)[2]	13.29 [-13.33; 39.90]	0.35
Medications (yes)	2.88 [-2.37; 8.14]	0.30

[1] Reference group: Pre-treatment measurement. [2] Reference group: Osteoarthritis severity (Grade 4).

Table S3. : Estimated model parameters for VAS Pain score in Group 2 (Responder group).

Variable	Regression coefficients [95% confidence interval]	p-value
Intercept	1.05 [-1.02; 3.13]	0.35
Measurement occasion 2 weeks[1]	-1.45 [-1.86; -1.04]	< 0.001
Measurement occasion 6 weeks[1]	-1.91 [-2.32; -1.50]	< 0.001
Measurement occasion 3 months[1]	-1.82 [-2.23; -1.41]	< 0.001
Measurement occasion 6 months[1]	-2.04 [-2.45; -1.63]	< 0.001
Measurement occasion 1 year[1]	-1.47 [-1.88; -1.06]	< 0.001
Age	0.02 [0.001; 0.04]	0.06
Sex (Male)	-0.20 [-0.72; 0.33]	0.49
BMI	0.06 [0.00; 0.11]	0.05
Duration Symptoms (Months)	0.01 [0.001; 0.02]	0.19
Number of treated joints (overall)	0.36 [-0.03; 0.75]	0.09
Booster (yes)	0.22 [-0.30; 0.74]	0.44
Osteoarthritis severity (Grade 2)[2]	-048 [-2.96; 1.99]	0.72
Osteoarthritis severity (Grade 3)[2]	-0.56 [-1.33; 0.21]	0.18
Medications (yes)	0.12 [-0.42; 0.67]	0.68

[1] Reference group: Pre-treatment measurement. [2] Reference group: Osteoarthritis severity (Grade 4).

Table S4. Estimated model parameters for VAS Pain score in Group 1 (Non-responder group).

Variable	Regression coefficients [95% confidence interval]	p-value
Intercept	5.95 [-1.42; 13.31]	0.25
Measurement occasion 2 weeks[1]	0.53 [-0.75; 1.82]	0.42
Measurement occasion 6 weeks[1]	0.34 [-0.94; 1.62]	0.61
Measurement occasion 3 months[1]	1.10 [-0.18; 2.38]	0.10
Measurement occasion 6 months[1]	3.07 [1.79; 4.35]	< 0.001
Measurement occasion 1 year[1]	1.02 [-0.26; 2.30]	0.13
Age	-0.01 [-0.06; 0.05]	0.86
Sex (Male)	1.06 [-0.24; 2.36]	0.25
BMI	-0.06 [-0.28; 0.16]	0.67
Duration Symptoms (Months)	0.03 [-0.10; 0.17]	0.71
Number of treated joints (overall)	-0.24 [-1.15; 0.67]	0.70
Booster (yes)	0.18 [-1.09; 1.45]	0.84
Osteoarthritis severity (Grade 3)[2]	-0.39 [-4.88; 4.09]	0.90
Medications (yes)	-1.34 [-2.39; -0.30]	0.08

[1] Reference group: Pre-treatment measurement. [2] Reference group: Osteoarthritis severity (Grade 4).

Table S5. Estimated model parameters for KOOS Symptoms score on the full cohort.

Variable	Regression coefficients [95% confidence interval]	p-value
Intercept	67.93 [52.42; 83.44]	< 0.001
Measurement occasion 3 months[1]	7.11 [4.91; 9.31]	< 0.001
Measurement occasion 6 months[1]	6.77 [4.58; 8.97]	< 0.001
Measurement occasion 1 year[1]	5.32 [3.13; 7.52]	< 0.001
Age	-0.02 [-0.18; 0.13]	0.76
Sex (Male)	1.90 [-1.86; 5.66]	0.34
BMI	-0.47 [-0.88; -0.06]	0.03
Duration Symptoms (Months)	0.02 [-0.06; 0.10]	0.69
Number of treated joints (overall)	-4.61 [-7.44; -1.79]	<0.001
Booster (yes)	1.03 [-2.70; 4.75]	0.60
Osteoarthritis severity (Grade 2)[2]	7.42 [-12.10; 26.95]	0.48
Osteoarthritis severity (Grade 3)[2]	0.69 [-5.00; 6.38]	0.82
Medications (yes)	2.79 [-1.07; 6.64]	0.18

[1] Reference group: Pre-treatment measurement. [2] Reference group: Osteoarthritis severity (Grade 4).

Table S6. Estimated model parameters for WOMAC Stiffness score on the full cohort.

Variable	Regression coefficients [95% confidence interval]	p-value
Intercept	112.57 [84.22; 140.92]	< 0.001
Measurement occasion 3 months[1]	5.66 [1.43; 9.89]	< 0.001
Measurement occasion 6 months[1]	6.96 [2.73; 11.19]	< 0.001
Measurement occasion 1 year[1]	2.59 [-1.63; 6.82]	0.23
Age	-0.32 [-0.59; -0.04]	0.03
Sex (Male)	0.16 [-6.71; 7.03]	0.97
BMI	-1.03 [-1.77; -0.28]	0.01
Duration Symptoms (Months)	-0.01 [-0.16; 0.13]	0.87
Number of treated joints (overall)	-5.46 [-10.62; -0.29]	0.05
Booster (yes)	-2.07 [-8.88; 4.73]	0.57
Osteoarthritis severity (Grade 2)[2]	36.49 [0.81; 72.17]	0.06
Osteoarthritis severity (Grade 3)[2]	2.58 [-7.82; 12.98]	0.64
Medications (yes)	2.88 [-4.17; 9.92]	0.44

[1] Reference group: Pre-treatment measurement. [2] Reference group: Osteoarthritis severity (Grade 4).

Table S7. Estimated model parameters for KOOS Daily Activities Score on the full cohort.

Variable	Regression coefficients [95% confidence interval]	p-value
Intercept	112.78 [90.03; 135.53]	< 0.001
Measurement occasion 3 months[1]	9.00 [5.92; 12.09]	< 0.001
Measurement occasion 6 months[1]	9.68 [6.60; 12.77]	< 0.001
Measurement occasion 1 year[1]	8.17 [5.09; 11.26]	0.23
Age	-0.37 [-0.59; -0.15]	< 0.001
Sex (Male)	0.90 [-4.61; 6.42]	0.76
BMI	-0.65 [-1.25; -0.06]	0.04
Duration Symptoms (Months)	-0.02 [-0.14; 0.09]	0.72
Number of treated joints (overall)	-4.69 [-8.84; -0.54]	0.04
Booster (yes)	0.41 [-5.05; 5.88]	0.89
Osteoarthritis severity (Grade 2)[2]	10.36[-18.29; 39.02]	0.50
Osteoarthritis severity (Grade 3)[2]	0.09 [-8.26; 8.44]	0.98
Medications (yes)	3.68 [-1.97; 9.34]	0.22

[1] Reference group: Pre-treatment measurement. [2] Reference group: Osteoarthritis severity (Grade 4).

Table S8. Estimated model parameters for KOOS Sport Score on the full cohort.

Variable	Regression coefficients [95% confidence interval]	p-value
Intercept	58.26 [4.57; 101.15]	0.06
Measurement occasion 3 months[1]	9.39 [2.67; 16.11]	0.01
Measurement occasion 6 months[1]	7.20 [0.48; 13.91]	0.04
Measurement occasion 1 year[1]	8.64 [1.92; 15.36]	0.01
Age	-0.23 [-0.73; -0.27]	0.41
Sex (Male)	9.55 [-3.14; 22.25]	0.18
BMI	-0.28 [-1.71; 1.14]	0.72
Duration Symptoms (Months)	-0.07 [-0.50; 0.35]	0.75
Number of treated joints (overall)	-5.77 [-16.11; 4.57]	0.32
Booster (yes)	11.69 [-0.69; 24.08]	0.10
Osteoarthritis severity (Grade 2)[2]	15.50 [-29.22; 60.22]	0.54
Osteoarthritis severity (Grade 3)[2]	12.99 [-4.22; 30.21]	0.18
Medications (yes)	-4.17 [-17.16; 8.38]	0.57

[1] Reference group: Pre-treatment measurement. [2] Reference group: Osteoarthritis severity (Grade 4).

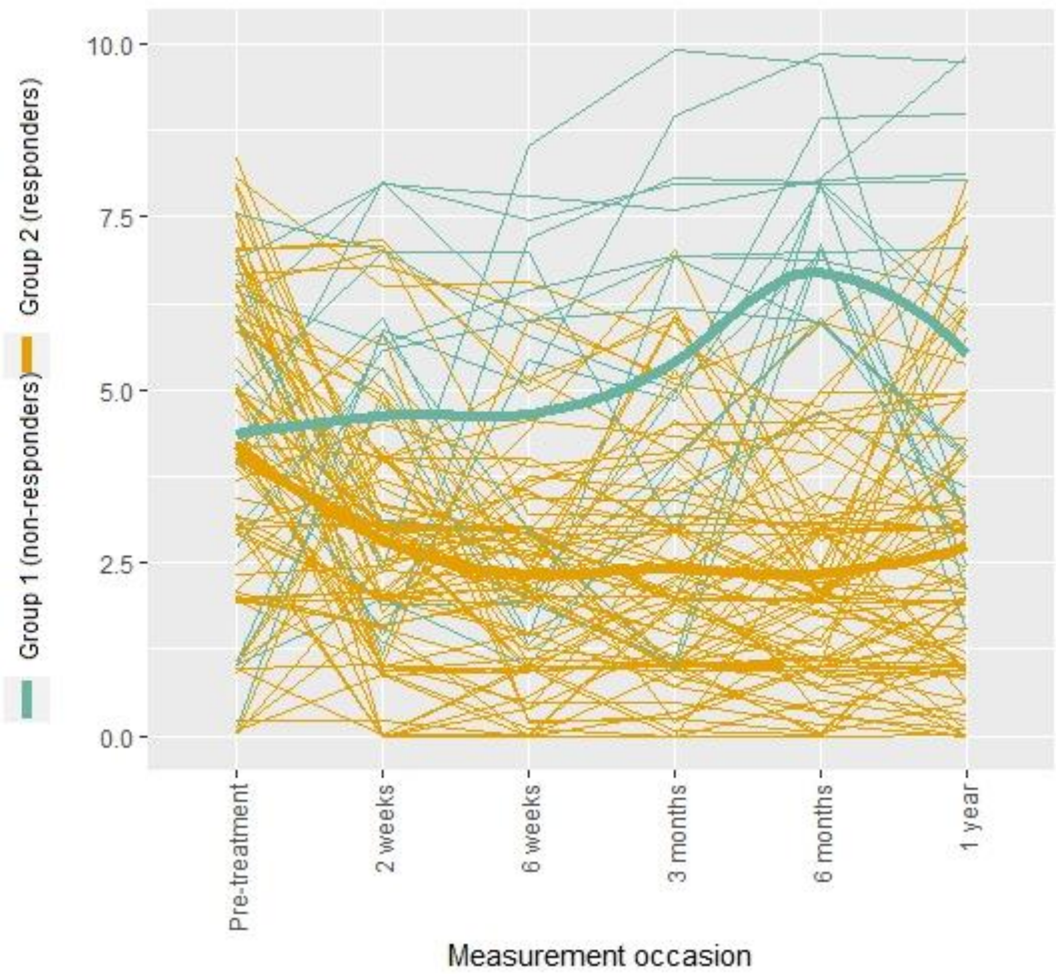

Figure S1. Individual trajectories of VAS Pain Score for therapy non-responders (Group 1) and therapy responders (Group 2).
